# Snare-based circumferential traction technique for endoscopic submucosal dissection of long circumferential early esophageal carcinoma

**DOI:** 10.1055/a-2098-0284

**Published:** 2023-07-27

**Authors:** Qin Lu, Zhi-qiang Du, Xiang-rong Zhou, Wei-hui Liu

**Affiliations:** 1 Department of Gastroenterology and Hepatology, Sichuan Academy of Medical Sciences and Sichuan Provincial People’s Hospital, Chengdu, China; 2 Department of Gastroenterology, Jianyang Peoples Hospital, Jianyang, China

Although tunneling dissection is reported to be effective in facilitating endoscopic submucosal dissection (ESD) of esophageal circumferential lesions 1 2 3 , endoscopists may lose visualization inside the narrow tunnel and it can be challenging to dissect the ridges between the tunnels 4 5 . Herein, we propose a snare-based circumferential traction technique to improve the long circumferential ESD of early esophageal cancer ( Video 1 ).

**Video 1** Snare-based circumferential traction-assisted endoscopic submucosal dissection of circumferential early esophageal carcinoma.


A 72-year-old man with long circumferential early esophageal cancer ( Fig. 1 a ) was referred for ESD treatment. After marking the lesion borders, anal and oral circumferential incisions were performed successively. The transparent cap-covered endoscope was withdrawn, and a snare was attached and reinserted into the esophagus. The pre-anchored snare was released from the scope and completely enveloped the incised mucosal flap with the help of an endoclip ( Fig. 1 b ). Under the snare-based circumferential traction force, the submucosal space was thoroughly exposed, and the submucosal fibers were stretched gently for efficient dissection ( Fig. 1 c ). As the direction of snare traction could be controlled not only by pulling, but also by pushing and rotating, the ESD procedure could be smoothly performed under clear vision ( Fig. 1 d ). After the lesion was fully dissected, a completely circumferential 5 cm surgical wound, without muscle injury, was presented ( Fig. 1 e ). The specimen was retrieved directly using the snare ( Fig. 1 f ). Histopathology revealed squamous cell carcinoma with invasion of the muscularis mucosa and negative margins.

**Fig. 1:**
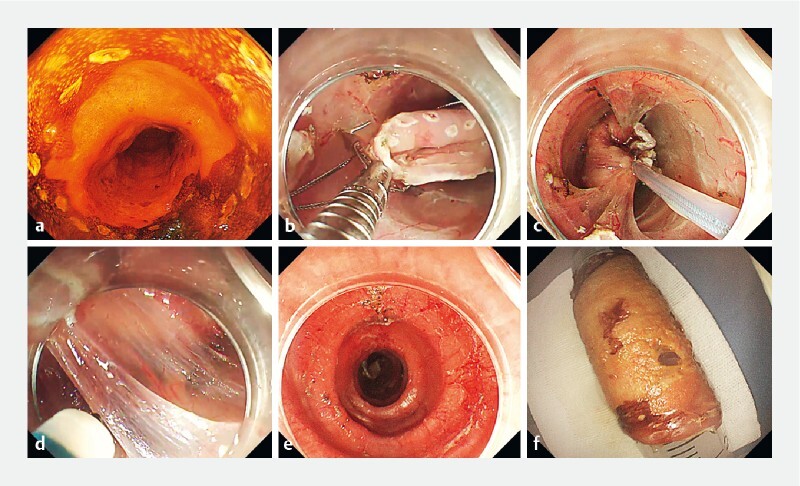
Schematic pictures of the snare-based traction-assisted endoscopic submucosal dissection for a long circumferential early esophageal carcinoma. **a** Chromoendoscopy demonstrated an unstained area measuring 5 cm in length, involving the esophageal circumference. **b** After circumferential incision, the pre-anchored snare was placed around the incised mucosal flap at the oral side using an endoclip. **c** The snare-traction approach allowed for good visibility and tension of the submucosa. **d** During submucosal dissection, the incised mucosa was pushed and rotated using the snare traction to expose the surgical field. **e** A long smooth surgical wound, without muscle damage, was presented. **f** The en bloc resected circumferential specimen was retrieved.

The snare-based circumferential traction technique not only creates enough space and effective tension for effective ESD, but also provides clear vision and enables dissection at a sufficient distance from the muscularis propria to allow safe ESD. In addition, this circumferential traction technique can stretch the submucosal fibers with perfect control for efficient dissection, making it unnecessary to repeatedly inject solution into the submucosa.

In summary, the novel circumferential traction-assisted ESD technique is simple and provides good visualization for tissue manipulation, and may become an attractive option in esophageal ESD of long circumferential early esophageal cancers.

Endoscopy_UCTN_Code_TTT_1AO_2AC

## Notes

Endoscopy E-Videos

https://eref.thieme.de/e-videos
*E-Videos* is an open access online section of the journal *Endoscopy* , reporting on interesting cases and new techniques in gastroenterological endoscopy. All papers include a high-quality video and are published with a Creative Commons CC-BY license. Endoscopy E-Videos qualify for HINARI discounts and waivers and eligibility is automatically checked during the submission process. We grant 100% waivers to articles whose corresponding authors are based in Group A countries and 50% waivers to those who are based in Group B countries as classified by Research4Life (see: https://www.research4life.org/access/eligibility/ ). This section has its own submission website at https://mc.manuscriptcentral.com/e-videos
